# Predictive clinical factors of cystoid macular edema in patients with Descemet’s stripping automated endothelial keratoplasty

**DOI:** 10.1038/s41598-017-07079-x

**Published:** 2017-08-07

**Authors:** Koji Kitazawa, Kanae Kayukawa, Koichi Wakimasu, Isao Yokota, Tsutomu Inatomi, Osamu Hieda, Kazuhiko Mori, Chie Sotozono, Shigeru Kinoshita

**Affiliations:** 1 Department of Frontier Medical Science and Technology for Ophthalmology, Kyoto Prefectural University of Medicine, Kyoto, Japan; 2 Department of Ophthalmology, Kyoto Prefectural University of Medicine, Kyoto, Japan; 3 Baptist Eye Institute, Kyoto, Japan; 4 Department of Biostatistics, Kyoto Prefectural University of Medicine, Kyoto, Japan

## Abstract

The purpose of this present study was to investigate predictive clinical factors associated with cystoid macular edema (CME) post Descemet’s stripping automated endothelial keratoplasty (DSAEK) in a large case series. Of 393 consecutive patients who underwent DSAEK at Baptist Eye Institute, Kyoto, Japan between July 2011 and November 2016, 241 patients without CME at the pre- or early-postoperative periods were enrolled. The occurrence of anatomic CME was prospectively examined via optical coherence tomography (OCT). Possible predictive clinical factors for CME were analyzed by multivariate logistic regression analysis. At 1-month post DSAEK, CME occurred in 27 (11.2%) of the 241 patients. Multivariate analysis revealed that primary angle closure glaucoma (PACG) was significantly associated with postoperative CME (odds ratio = 6.4, *P* = 0.04). The findings of this study revealed that DSAEK in patients with PACG showed a high incidence of CME, thus indicating that they should undergo a careful postoperative observation of the macula via OCT.

## Introduction

Bullous keratopathy (BK) is caused by corneal endothelial dysfunction, and leads to corneal swelling that ultimately results in a severe loss of vision. Worldwide, BK is the primary causative disease and leading indication for patients who undergo corneal transplantation^1^.

We previously reported that in a small group of glaucoma patients who underwent Descemet’s stripping automated endothelial keratoplasty (DSAEK), a high degree of postoperative cystoid macular edema (CME) occurred^2^. First reported by Irvine in 1953^3^, CME often occurs post cataract surgery with no complications^4–7^, and leads to transient visual impairment^8^. It is believed that CME is also one of the important factors that induces visual impairment in patients who undergo penetrating keratoplasty (PKP)^9, 10^ and endothelial keratoplasty^11, 12^. However, the causative clinical factors associated with developing CME post DSAEK have yet to be fully elucidated.

Optical coherence tomography (OCT), an imaging system that provides precise findings of the retina, allows for a more accurate diagnosis of retinal abnormalities such as CME, macular edema due to retinal vein occlusion and diabetic retinopathy, macular epiretinal membrane, and age-related macular degeneration (AMD)^12–14^. In fact, OCT is so precise that it can be used to diagnose macular diseases in patients undergoing DSAEK in whom CME might be overlooked at the pre- and postoperative periods.

Thus, the purpose of this present study was to prospectively investigate causative clinical factors in regard to anatomic CME post DSAEK via OCT on a large number of patients by use of multivariate logistic regression analysis.

## Results

### Included patients

**Figure 1:**
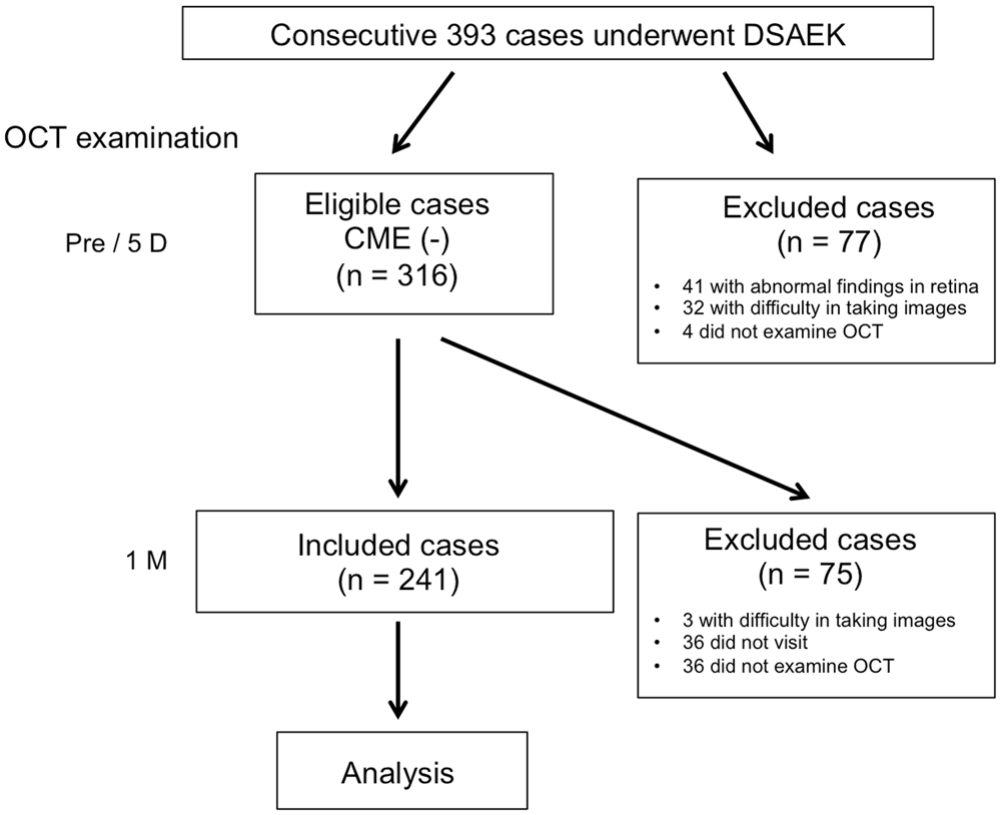
Flow chart illustrating the inclusion and exclusion criteria in this prospective study.

Of 393 consecutive patients who underwent DSAEK, 241 patients [91 males and 150 females, mean age: 71.7 ± 12.1 (mean ± standard deviation) years] who met the study criteria were enrolled. A flow chart illustrating the inclusion and exclusion criteria is shown in Fig. 1.

### Incidence of postoperative CME

**Figure 2:**
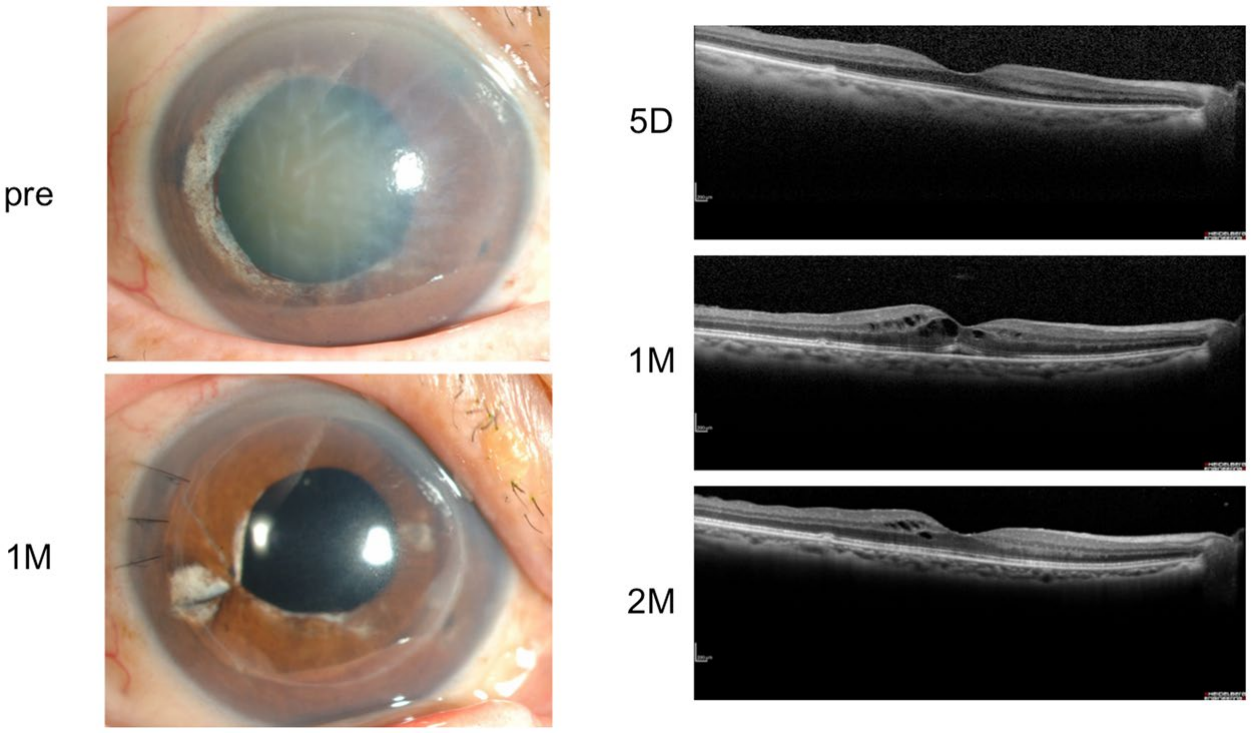
Representative case. The right eye of a 71-year-old female who had undergone argon-laser iridectomy for primary angle-closure glaucoma (PACG) in 2007. Since that time, corneal endothelial dysfunction had gradually progressing, resulting in bullous keratopathy (BK) and a visual acuity (VA) of 20/2000. Thus, she was treated by Descemet’s stripping automated endothelial keratoplasty combined with cataract surgery in July 2013. Her dilated pupil was reconstructed by iris suture. Postoperative inflammation was not severe, and the findings of an optical coherence tomography (OCT) examination at 5-days postoperative were normal, although the OCT image was not clear due to severe BK and cataract prior to surgery. At her 1-month follow-up visit, cystoid macular edema (CME) was observed and a 2-times daily administration of bromfenac eye drops was added to the routine postoperative treatment. The following month, the CME decreased and her VA was 20/20.

In all of the 241 enrolled patients, the macula was found to be normal prior to DSAEK surgery or at 5-days postoperative, however, OCT examination of the macula at 1- to 2-months postoperative showed anatomic CME in 27 of the 241 patients (11.2%). Of the primary causes of BK, glaucoma-related eyes accounted for the highest incidence of postoperative CME (n = 14, 18.2%), while Fuchs endothelial corneal dystrophy (FECD) accounted for the lowest incidence of postoperative CME (n = 1, 3.6%). A summary of the incidence of postoperative CME is shown in Table 1. Of 12 primary angle closure glaucoma (PACG) cases, all had previously experienced an acute glaucoma attack, and 8 of those cases (4 CME+ cases and 4 CME− cases) had undergone argon laser iridotomy (ALI). Representative case with PACG is shown in Fig. 2.

**Table 1:** Incidence of postoperative CME according to the primary causes of bullous keratopathy.

The primary causes of bullous keratopathy	CME+, No.	Total, No.	Incidence of CME (%)
Glaucoma-related eyes	(14)	(77)	18.2
PACG	6	12	50.0
PEG	2	16	12.5
POAG	6	49	12.2
Aphakia or pseudophakia	4	43	9.3
Prophylactic argon laser iridotomy	4	59	6.8
Fuchs endothelial corneal dystrophy	1	28	3.6
Others	4	34	11.8
Total	27	241	11.2

CME: cystoid macular edema, PACG: primary angle-closure glaucoma, PEG: pseudoexfoliation glaucoma, POAG: primary open-angle glaucoma, No: number.

### Predictive clinical factors of CME

In order to predict postoperative CME, we next assessed which clinical factors, i.e. patient age, primary causes, extensive iris damage, combination of lens surgeries, rebubbling of air for the detachment of the corneal graft, previous corneal transplantation, pretreatment with steroid eye drops including 0.1% betamethasone and 0.1% fluorometholone, or a past history of mild anterior uveitis and diabetes, which could affect the postoperative intraocular inflammation, were associated with CME post DSAEK. Multivariate analysis showed that PACG alone was significantly correlated with the occurrence of postoperative CME [CME+: 6 cases (22%), CME−: 6 cases (3%), odds ratio (OR) = 6.4, *P* = 0.04] (Table 2). Contrary to our previous expectations, which have shown the blood-aqueous barrier breakdown in eyes with endothelial decompensation after ALI^13, 14^, prophylactic ALI did not identify in the risk factor for CME [CME+: 4 cases (15%), CME−: 55 cases (26%)]. Unexpectedly, the combination of lens surgeries [CME+: 8 cases (30%), CME−: 64 cases (30%)], rebubbling of air [CME+: 4 cases (15%), CME−: 14 cases (7%)], pretreatment with steroid eye drops [CME+: 17 cases (63%), CME−: 132 cases (62%)], and a past history of uveitis [CME+: 2 cases (7%), CME−: 17 cases (8%)] was found not to be statistically associated with the incidence of CME, and there seemed to be a negative association between diabetes history and postoperative CME [CME+: 4 cases (15%), CME−: 61 cases (29%)]. Among the patients in whom CME occurred, there was no episode of using eye drops of prostaglandin analogs for postoperative intraocular pressure (IOP) elevation.

**Table 2:** Predictive clinical factors in patients with cystoid macular edema.

Clinical Factors	Multivariate analysis		
	Odds ratio	95% CI	*P*-value
Age at surgery			
>65 years old	1.1	0.4–3.3	0.92
Primary causes of bullous keratopathy (Ref: others)			
PACG	6.4	1.1–40.8	0.04
PEG	1.3	0.2–8.9	0.78
POAG	1.0	0.2–5.0	0.99
Aphakia or pseudophakia	0.8	0.2–4.1	0.78
Prophylactic argon laser iridotomy	0.7	0.1–3.9	0.67
Fuchs endothelial corneal dystrophy 0.3 0.0–2.7 0.31	0.3	0.0–2.7	0.31
Iris damage			
>2 quadrants	2.3	0.8–6.2	0.11
Combined with cataract surgery			
Yes	1.4	0.5–4.1	0.57
Rebubbling			
Yes	2.0	0.5–7.6	0.33
Previous corneal transplantation			
Yes	1.0	0.2–3.7	0.97
Pretreatment with topical steroid			
Yes	1.3	0.5–3.5	0.60
Past history of uveitis			
Yes	0.8	0.1–4.1	0.85
Past history of Diabetes			
Yes	0.4	0.1–1.2	0.11
Total			

CME: cystoid macular edema, CI: confidence interval, PACG: primary angle-closure glaucoma, PEG: pseudoexfoliation glaucoma, POAG: primary open-angle glaucoma.

### Analysis of the association between iris damage and primary causes

Postoperative CME found to be more frequently occurred in patients with PACG, however, the reason for that was not clarified. Multivariate analysis revealed that odd ratio in extensive iris damage was relatively high (OR = 2.3), yet, it was not significantly associated with the occurrence of CME post DSAEK. Thus, to examine what iris damage was associated, multivariate analysis between primary causes and extensive iris damage adjusting for age, combination of lens surgeries, previous corneal transplantation, past history of mild anterior uveitis and diabetes, was performed. Multivariate analysis revealed that PACG was significantly associated with iris damage (OR = 5.0, *P* = 0.04), yet not significant associations between iris damage, and prophylactic ALI-induced BK and aphakia/pseudophakia-induced BK (Table 3).

**Table 3:** Analysis of the association between iris damage and primary causes.

Primary causes	Adjusted analysis^†^		
	Odds ratio	95% CI	P-value
Glaucoma-related eyes			
PACG	5.0	1.1–25.0	0.04
PEG	—	—	—
POAG	1.9	0.6–6.1	0.26
Prophylactic argon laser iridotomy	0.4	0.1–1.2	0.11
Aphakia/pseudophakia	1.1	0.3–3.7	0.84
Fuchs endothelial corneal dystrophy	—	—	—
Others			
Total			

CI: confidence interval, PACG: primary angle-closure glaucoma, PEG: pseudoexfoliation glaucoma, POAG: primary open-angle glaucoma. ^†^Adjusting for age, combination of lens surgeries, previous corneal transplantation, past history of mild anterior uveitis and diabetes. We excluded PEG and Fuchs endothelial corneal dystrophy from adjusted analysis because there were a few patients with extensive iris damage.

## Discussion

In this present study, our findings demonstrated that anatomic CME post DSAEK occurred in 11.2% of the enrolled patients. Previous studies conducted in the United States reported that CME post DSAEK was observed in 4% of the patients, which is relatively low compared with our findings^11^. Our findings of CME post DSAEK in patients with FECD was 3.6% (i.e. also relatively low), thus suggesting that the low incidence of postoperative CME in the previous reports may be somewhat related to the dominance of patients with FECD. Of 77 glaucoma-related patients who underwent DSAEK for BK, postoperative CME occurred in 14 (18.2%), indicating that glaucoma-related eyes accounted for the highest incidence of postoperative CME, and it was very similar to the findings in our previous small case series^2^.

In this present study, we investigated the clinical factors of CME post DSAEK, and the association between CME and glaucoma-related eyes (i.e. PACG, pseudoexfoliation glaucoma (PEG) and POAG, respectively) in more detailed analysis. According to multivariate analysis, extensive iris damage, a combination of cataract surgery, previous corneal transplantation, rebubbling of air, pretreatment with steroid eye drops, a past history of uveitis and diabetes, which could affect postoperative inflammation, were not significantly associated with postoperative CME. We previously reported that ALI for shallow eyes caused the breakdown of the blood-aqueous barrier^14^, thereby resulting in the increase of inflammation, and could cause the development of postoperative CME. However, in this present study, DSAEK for prophylactic ALI-induced BK did not increase the incidence of postoperative CME. Conversely, of 12 PACG cases, including 8 PACG cases that underwent ALI, 6 PACG cases had postoperative CME, and PACG was significantly associated with the occurrence of postoperative CME, suggesting that there may be other factors that can lead to the development of CME post DSAEK.

We speculated that severe iris damage appeared to disrupt the blood-aqueous barrier, resulting in postoperative CME after DSAEK, because patients with PACG caused the dilation and deformation of pupils due to acute angle closure attack and often underwent multiple glaucoma surgery. Recent paper in regard to the association between iris damage and inflammation in aqueous humor has supported our hypothesis^15^. In fact, the extensive iris damage beyond 2 quadrants was found in none of the FECD patients and in 7 (11.0%) of the patients with prophylactic ALI, yet it was found in 26 (33.8%) of the glaucoma patients, especially the PACG patients (58.3%). This might be one possible explanation of why FECD and prophylactic ALI accounted for the low incidence of postoperative CME (3.6% and 6.8%, respectively), while glaucoma-related eyes accounted for the highest incidence of postoperative CME (18.2%). However, multivariate analysis of clinical factors showed that there was no significant association between severe iris damage and postoperative CME, while PACG alone statistically accounted for the highest occurrence of postoperative CME, even after adjusting for covariate factors including iris damage (OR = 6.4, *P* = 0.04). Those findings suggested that in patients with PACG, the environment inside the anterior chamber was extremely altered, regardless of the presence or absence of iris damage. It has been reported that in the anterior chamber of patients with BK, many inflammatory cytokines are elevated^16^, thus suggesting that factors other than iris damage can alter the environment inside the anterior chamber in patients with PACG, and that some inflammatory factors inside the anterior chamber can lead to move to the posterior precortical vitreous pocket through a cannel connection the Cloquet’s canal, ultimately inducing the development of postoperative CME^17^.

A postoperative steroid treatment regimen appears to be strongly associated with the development of CME. In fact, intensive topical steroid therapy conducted during the first postoperative week reduced the incidence of CME, at least after triple-DMEK surgery^18^. Thus, the use of steroids might have led to lower CME rates in these patients. Our postoperative steroid regimen was a 4-times-daily administration of 0.1% betamethasone eye drops, similar to that described in the study by Suh and associates^11^. In addition, each patient received a systemic dose of 125-mg methylprednisolone 1-hour prior to surgery in order to reduce early postoperative inflammation. In some of the patients, pretreatment with topical steroids was performed because moist corneal epithelium due to BK can cause the epithelium to become fragile, and studies have shown that epithelial cell loss repeatedly occurs, thereby leading to the increase of proinflammatory cytokines^16, 19^ that might be one of the causative factors leading to CME. Thus, we compared the rate of CME in patients who did or did not undergo topical steroid therapy, yet discovered that there was no significant difference between the two groups. Further investigation is needed to more fully elucidate which postoperative treatment regimens can successfully prevent the development of CME.

In conclusion, the findings of this present study reveal that after treating patients with PACG, strict attention should be paid to the possible occurrence of CME via OCT of the macula, which can elucidate imperceptible changes. We are planning to conduct the therapeutic interventional study in the future.

## Patients and Methods

This prospective study was approved by the Institutional Review Board of Baptist Eye Institute, Kyoto, Japan (approval number: 1604). In accordance with the tenets set forth in the Declaration of Helsinki, written informed consent was obtained from all patients prior to their involvement in the study. Clinical trial registration was obtained from UMIN (R000028644 UMIN000024892, http://www.umin.ac.jp/english/).

This study involved 393 consecutive patients who underwent DSAEK surgery at the Baptist Eye Institute from July 2011 to September 2016. In each patient, OCT (3D OCT 1000; Topcon Corporation, Tokyo, Japan, or SPECTRALIS^®^; Heidelberg Engineering, Heidelberg, Germany) examination was used to assess CME. Patient inclusion and exclusion criteria are shown in Fig. 1 and were as follows: Patients without CME at the preoperative period were included in this study. Of the patients in whom a clear OCT image could not be obtained at the preoperative period due to severe BK, patients without CME at early postoperative period (i.e. 5-days postoperative) were included. Patients excluded from the study were (1) those with retinal abnormalities, such as AMD, macular edema due to retinal vein occlusion, and macular epiretinal membrane, (2) those in whom a clear OCT image was difficult to obtain at the pre- and early postoperative period, and (3) those who did not undergo OCT examination or who did not visit our hospital at the appropriate time. In accordance with the above criteria, 241 of the original 393 cases were ultimately included in this study.

Primary causes for the BK were as follows; glaucoma-related eyes including PACG, POAG, and PEG, post cataract surgery (i.e. pseudophakia or aphakia), post ALI as prophylactic treatment for primary angle closure, and FECD. Other causes included iridocorneal endothelial syndrome, corneal endotheliitis including Cytomegalovirus corneal endotheliitis^20^, posterior polymorphous corneal dystrophy, Axenfeld-Rieger syndrome, chemical burn, birth-related injury, toxic anterior segment syndrome, post radial keratotomy, post PKP in patients with keratoconus, syphilitic keratitis, herpetic stromal keratitis and macular corneal dystrophy, shallow eyes without ALI (including 1 patient with peripheral iridectomy), and unknown causes. Seventy-two patients had undergone DSAEK simultaneously combined with intraocular lens (IOL) implantation, including lens insertion, lens exchange, and lens suturing, and 33 patients who had previously undergone DSAEK or PKP had failed grafts. Eighteen patients had undergone rebubbling for detachment of the corneal graft, and 149 patients had received topical steroid therapy including 0.1% betamethasone and 0.1% fluorometholone prior to DSAEK. Nineteen patients had a past history of mild anterior uveitis, including 4 patients with a past history of cytomegalovirus corneal endotheliitis. Sixty-five patients had systemically diabetes. Pre-existing iris damages were assessed by slit-lamp examination.

### Surgical technique

Donor corneas were obtained from SightLife^™^ Eye Bank (Seattle, WA, USA), and all of the DSAEK flaps were prepared by SightLife^™^ prior to shipment to Japan. Each patient was administered either general or retrobulbar anesthesia. After an anterior chamber maintainer was set up, the Descemet’s membrane at a 7.00-mm diameter area of the central cornea was removed using a reverse Sinskey hook (Bausch & Lomb, Rochester, NY, USA). The donor epithelium was scraped off when the Descemet’s membrane or the inside of the anterior chamber was poorly visible due to corneal edema. A corneal graft button 7.25- to 9.0-mm in diameter was cut for transplantation by use of a Barron Vacuum Punch (Barron Precision Instruments, LLC., Grand Blanc, MI, USA). Using a Busin glide, a DSAEK flap was then inserted into the anterior chamber through a 4-mm corneal incision at the temporal limbus. The flap was then placed stromal side up and the anterior chamber was filled with air to produce high IOP to firmly attach the graft to the host cornea. After 10 minutes, the IOP was adjusted to over 10 to 20 mmHg by adding and aspirating air and BSS PLUS^®^ Irrigating Solution (Alcon Inc., Hünenberg, Switzerland) using a 30-gauge needle. In the patients with combined cataract surgery, the scleral tunnel was made 2.5-mm wide at the superior position. An IOL was then implanted by use of an IOL insertion device (Alcon) after the cataract surgery via phacoemulsification and aspiration.

### Postoperative management

As per the protocol reported in our previous study^2^, following DSAEK, each patient received a systemic dose of 125-mg methylprednisolone 1-hour prior to surgery, and then a systemic dose of 4-mg betamethasone for 2-days postoperative followed by 1-mg betamethasone for an additional 5 days. In addition, a topical application of 0.3% gatifloxacin and 0.1% betamethasone eye drops was administered 4-times daily, and at 6-months post DSAEK, the 0.1% betamethasone eye drops were switched to 0.1% fluorometholone eye drops instilled 2- to 4-times daily. For patients with general problems such as poor control of diabetes, renal failure, etc., the systemic dose of steroid was appropriately adjusted. Anti-glaucoma drops were stopped in all patients at the time of surgery.

### Clinical examination

In each patient, OCT (Topcon 3D OCT-1000 and SPECTRALIS^®^) was used to examine the macula prior to surgery and at 5-days and 1- to 2-months postoperative, and the presence and absence of CME was then assessed. In cases with CME at 1-month postoperative, macula examination was continued. The fundus was examined by multiple scans (i.e. both vertical and horizontal). Fundus examination was also carefully performed via retinoscopy. Postoperative CME was defined as anatomic CME, because it is difficult to assess the existence of symptoms at the early post-DSAEK period due to the fact that corneal swelling still remains and the patients often hardly notice any symptomatic disturbance.

### Statistical analysis

Statistical analyses were performed using JMP^®^ Pro ver. 12.1.0 statistics software (SAS, Cary, NC, USA). To identify causative clinical factors associated with the incidence of postoperative CME, analyses was performed with multivariate logistic regression analysis that included age at surgery, primary causes, extensive iris damage, combination with lens surgery, previous corneal transplantation, past history of mild anterior uveitis and diabetes as covariates. The incidence OR with 95% confidence intervals was calculated. To identify the association between extensive iris damage and primary causes, ORs were estimated from logistic model including each primary cause and the adjusted factors such as age at surgery, combination with lens surgery, previous corneal transplantation, past history of mild anterior uveitis and diabetes. We excluded PEG and Fuchs endothelial corneal dystrophy from adjusted analysis because there were a few patients with extensive iris damage. A *P*-value of less than 0.05 was considered statistically significant.
